# Supplementary material for: A callosal biomarker of behavioral intervention outcomes for autism spectrum disorder? A case-control feasibility study with diffusion tensor imaging
Source: PLoS One. 2022 Feb 3;17(2):e0262563. doi: 10.1371/journal.pone.0262563 (PMC8812884; doi:10.1371/journal.pone.0262563)
Supplement: S1 Appendix — (PDF) [file pone.0262563.s001.pdf]

AD HOC AUTISM SEVERITY QUESTIONNAIRE

a. When first diagnosed

1. Verbal communication deficits[1]	[1] Severe	[2] Marked	[3] noticeable
2. Nonverbal communication deficits[2]	[1] Severe	[2] Marked	[3] noticeable
3. Initiating social interactions[3]	[1] Very limited	[2] Limited	[3] mild limitation
4. Responding to others[4]	[1] Very minimal	[2] Reduced	[3] generally good
5. Typical communication	[1] Few words	[2] Simple sentences	[3] Full sentences
6. Difficulty coping with change or switching between activities	[1] Extreme	[2] Frequent	[3] In some contexts

TOTAL SCORE[5] |___|

b. Currently

1. Verbal communication deficits[1]	[1] Severe	[2] Marked	[3] noticeable
2. Nonverbal communication deficits[2]	[1] Severe	[2] Marked	[3] noticeable
3. Initiating social interactions[3]	[1] Very limited	[2] Limited	[3] mild limitation
4. Responding to others[4]	[1] Very minimal	[2] Reduced	[3] generally good
5. Typical communication	[1] Few words	[2] Simple sentences	[3] Full sentences
6. Difficulty coping with change or switching between activities	[1] Extreme	[2] Frequent	[3] In some contexts

TOTAL SCORE[5] |___|

Notes

[1] Severe: few intelligible words; Marked: uses simple sentences; Noticeable: may struggle with to-and-fro conversation, but is able to use complex sentences.

[2] Severe: rarely uses or responds to non-verbal cues; Marked: uses a restricted range of gestures, which are markedly odd; Noticeable: communicates using gestures and can follow non-verbal cues.

[3] Very limited: almost never initiates; Limited: sometimes initiates, often needs prompting; Mild limitation: rarely needs prompting (e.g., will greet familiar people).

[4] Very minimal: responds to others only if prompted; Reduced: interaction is narrowly limited to special interests; Generally good: can engage in conversations but has difficulties in maintaining to-and-fro conversations.

[5] Mark a single choice per item. Compute the total score by adding the choice score across items. Scale scores range, 6 to 18.
