# Supplementary material for: A callosal biomarker of behavioral intervention outcomes for autism spectrum disorder? A case-control feasibility study with diffusion tensor imaging
Source: PLoS One. 2022 Feb 3;17(2):e0262563. doi: 10.1371/journal.pone.0262563 (PMC8812884; doi:10.1371/journal.pone.0262563)
Supplement: Attachment — Submitted filename: PONE-D-21-30729.docx [file pone.0262563.s003.docx]

Virues-Otega et al. evaluated white matter integrity between subjects with ASD that had or had not received PMBI. Overall, the manuscript is well-written, and the findings appear robust. However, I do have some concerns.

- The major drawback of this study is the small sample size. Furthermore, longitudinal data is preferable to evaluate the effect of treatment/behavioral intervention.
- Why the authors only evaluated FA despite its unspecificity? While AD and RD are assumed to represent axon and myelin integrity, respectively, I suggest including MD, AD, and RD in the analysis.
- Were there any significant differences in intelligence between groups that might have affected the results of this study?
- Multiple comparisons correction should be applied in the ROI analysis.

Minor comments:

- This study included not only subjects with autism but also subjects with Asperger syndrome and pervasive developmental disorder; please change the title “autism” —> “autism spectrum disorder.”
- Please use the consistent term “autism spectrum disorder” throughout the manuscript.
- The introduction of this manuscript is too long (4.5 pages) and unfocused.
- Please define all abbreviations on their first use in text, such as DSM, ADOS, and ADIS.
- Page 8, line 176. “Overall, 11 participants received PMBI, while 14 had received other services,” please describe “other services” more precisely and explain will or will not this service affect the results.
- Please provide the p-values in table 1.
- Page 7, line 166: “The final sample of 26 individuals included 17 subjects with ASD, four with Asperger syndrome, and four with pervasive developmental disorder…” Did the total number of participants calculated correctly?
- Please state the duration of PMBI.
- Please describe the statistical analysis of TBSS. Did the authors include age, gender, and intracranial volume as covariates?
